# The Role of MARCKS in Metastasis and Treatment Resistance of Solid Tumors

**DOI:** 10.3390/cancers14194925

**Published:** 2022-10-08

**Authors:** Chun-Lung Chiu, Hongjuan Zhao, Ching-Hsien Chen, Reen Wu, James D. Brooks

**Affiliations:** 1 Department of Urology, School of Medicine, Stanford University, Stanford, CA 94304, USA; 2 Department of Internal Medicine, Division of Pulmonary and Critical Care Medicine, Center for Comparative Respiratory Biology and Medicine, School of Medicine, University of California Davis, Davis, CA 95616, USA; 3 Department of Internal Medicine, Division of Nephrology, University of California Davis, Davis, CA 95616, USA; 4 Department of Anatomy, Physiology and Cell Biology, School of Veterinary Medicine, University of California Davis, Davis, CA 95616, USA

**Keywords:** MARCKS, cancer metastasis, cancer stemness, treatment resistance

## Abstract

**Simple Summary:**

Cancer metastasis is a critical event in the progression of solid tumors and is invariably associated with adverse outcomes and mortality. Understanding novel mechanisms or molecules that promote cancer metastasis will facilitate the development of new strategies for cancer treatment. Recently, MARCKS has been studied extensively in several cancers and has been implicated in tumor progression and metastasis. This review summarizes recent advances in the understanding of MARCKS on cancer metastasis, stemness, and therapeutic resistance and provides prospects on targeting MARCKS therapeutically. Specifically, we review the molecular mechanisms and multiple signaling pathways by which MARCKS contributes to the progression and metastasis in solid tumors.

**Abstract:**

The myristoylated alanine-rich C-kinase substrate (MARCKS) is a membrane-associated protein kinase C (PKC) substrate ubiquitously expressed in eukaryotic cells. MARCKS plays important roles in multiple cellular processes, including cell adhesion and motility, mucin secretion, exocytosis, and inflammatory response. Aberrant MARCKS signaling has been observed in the development and progression of multiple cancer types. In addition, MARCKS facilitates cancer metastasis through modulating cancer cell migration and invasion. Moreover, MARCKS contributes to treatment resistance, likely by promoting cancer stem cell renewal as well as immunosuppression. In this review, we describe MARCKS protein structure, cellular localization, and biological functions. We then discuss the role of MARCKS in cancer metastasis as well as its mechanisms of action in solid tumors. Finally, we review recent advances in targeting MARCKS as a new therapeutic strategy in cancer management.

## 1. Introduction

Metastasis, defined by disseminated cancer cells at sites distant from the primary tumor, is the principal cause of cancer death, as shown by the stark differences in 5-year survival rates for localized compared to metastatic disease [1]. Studies have shown that large numbers of disseminated tumor cells are released from the primary tumor in the early stages of cancer growth. However, only a small fraction of these cells are able to colonize distant foreign tissue sites, adapt to a relatively inhospitable microenvironment and then progress from micro- to macro-metastatic disease [2]. These metastasis-initiating cells often possess stem-like properties, allowing them to undergo epithelial-mesenchymal transitions, enter slow-cycling states for dormancy, evade immune surveillance, establish supportive interactions with organ-specific niches, and co-opt systemic factors for growth and resistance to anticancer therapies [3,4,5]. A growing body of evidence implicates the myristoylated alanine-rich C-kinase substrate (MARCKS), and the highly homologous MARCKS-like protein 1 (MARCKSL1) in cancer migration and metastasis. Both MARCKS and MARCKSL1 are activated by phosphorylation, suggesting that MARCKS-targeted therapies could be used to treat cancer metastasis [6,7,8,9,10,11,12,13]. Our understanding of the molecular mechanisms underlying the role of MARCKS in promoting cancer metastasis and therapeutic resistance is still incomplete; however, much progress has been made [14,15]. In hematological malignancies, MARCKS expression and phosphorylation have been implicated in treatment resistance and are associated with disease-specific mortality [16]. However, whether increased MARCKS confers treatment resistance in solid tumors remains unclear. In this review, we discuss the structure, localization, and function of MARCKS, describe the role and mechanisms of MARCKS in potentiating cancer metastasis and review whether MARCKS contributes to treatment resistance in solid tumors.

## 2. Protein Structure and Cellular Localization of MARCKS

MARCKS, an actin filament crosslinking protein, has a 32 kDa molecular weight that was originally identified as an 87 kDa protein substrate for Protein Kinase C (PKC) due to its anomalous molecular behavior and is ubiquitously expressed in eukaryotic cells [16,17,18]. This rod-shaped protein contains three distinct evolutionarily conserved regions: the N-terminal myristoylated domain (NMD), the multiple homology 2 domain (MH2), and the phosphorylation site domain (PSD) (also known as effector domain (ED)) [19]. The NMD recognized by N-myristoyl transferase mediates the insertion of the myristoyl moiety into the hydrophobic lipid bilayer of the plasma membrane [20], while the MH2 domain interacts with actin and contains a potential dimerization motif [18]. The highly positively charged PSD is crucial for the functionality of MARCKS and the source of its ability to electrostatically bind to phosphatidylinositol bisphosphate (PIP2), a docking site on the inner leaflet of the plasma membrane and a direct activator of numerous membrane proteins [21]. Phosphorylation by PKC within MARCKS PSD at Ser159, Ser163, and Ser170 directly or through RhoA/ROCK at Ser159 [22] or calcium-dependent calmodulin-binding reduces MARCKS binding to PIP2 and leads to MARCKS release from the plasma membrane into the cytoplasm, where it acts as a key regulatory protein [23].

## 3. Biological Functions of MARCKS

The biological functions of MARCKS primarily depend on its phosphorylation-dephosphorylation status, which in turn determines its membrane vs. cytosolic localization where it interacts with its two main binding partners, actin and PIP2 [14,15]. At the plasma membrane, phosphorylated MARCKS directly binds to and cross-links filamentous actin to modulate cytoskeletal structure in critical biological processes such as wound healing, morphogenesis, embryogenesis, and metastasis [24,25]. In addition, MARCKS has been proposed to link secretory granules to the cytoskeletal actin and myosin for exocytosis [26]. Moreover, MARCKS sequesters PIP2 at lipid rafts in the cell membrane in various cell types, including neutrophils [27,28], macrophages [29], fibroblasts [30], and hepatic stellate cells [31], to regulate cell motility and chemotaxis. By regulating PIP2 and its downstream secondary messengers such as inositol-1,4,5-trisphosphate (IP3) and diacylglycerol (DAG) [32,33], MARCKS indirectly modulates multiple cellular processes, including cell migration, membrane trafficking, mitosis, vesicular trafficking, receptor endocytosis, exocytosis, and cytoskeletal reorganization [34]. Finally, MARCKS mediates the inflammatory response through the regulation of cell migration and inflammatory cytokines in macrophages and neutrophils [19,35,36,37].

## 4. MARCKS in Cancer Metastasis

**Figure 1:**
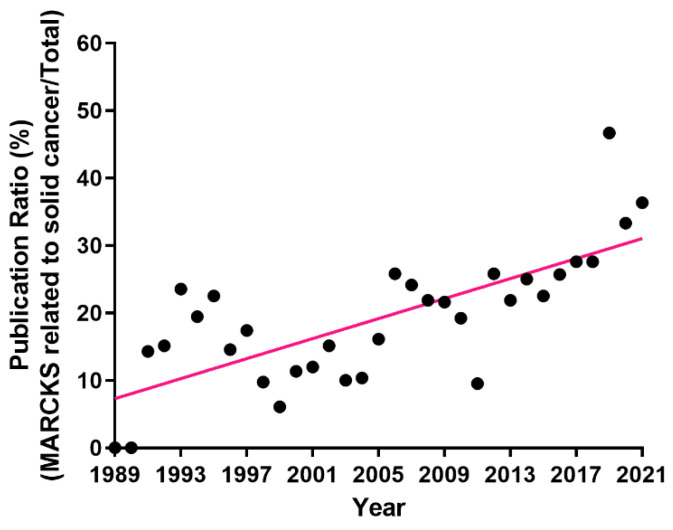
The publication trend of MARCKS research related to solid tumors in PubMed from 1989 to 2021. The black dots represent the publication ratio (%) of each year in which the publications of MARCKS research related to solid tumors were divided by the total publications of MARCKS research. The red line demonstrates the trend of the non-linear regression fit curve analyzed from each year’s publication ratio by GraphPad Prism (v6.07).

MARCKS signaling has been implicated in promoting cancer progression and metastasis in several solid malignancies through the interactions of its highly conserved effector domain with other crucial proteins such as actin [38], PKC, and AKT [14]. Since MARCKS was first implicated in solid tumors [39], the number of publications has grown rapidly, and the ratio of solid tumor-related publications to total MARCKS publications has increased dramatically from 1989 to 2021 (Figure 1).

In lung cancer, MARCKS phosphorylation is correlated with advanced stage and lymph node metastasis and predicts shorter survival [40,41]. Elevated MARCKS phosphorylation enhances migration and invasion of lung cancer cells in vitro and metastasis in vivo [9], while a MARCKS-inhibiting peptide attenuates cell growth, migration, and invasion and reduces metastasis in both subcutaneous and orthotopic xenograft models, likely through modulating NF-κB signaling [42,43,44]. In addition, an inhaled MARCKS inhibitor, BIO-11006, demonstrated an improvement of the overall response rate in patients [45]. In addition, MARCKS overexpression has been observed in aggressive subtypes of breast cancer, i.e., basal-like and HER2 subtypes [46], and is also associated with tumor grade, presence of metastases, and poor survival in male breast cancer and inflammatory breast cancer [47,48,49], possibly through increased MARCKS binding to Tob which decreases binding of Tob with ErbB2 and subsequent activation of ErbB2 signaling [50,51]. In renal cell carcinoma (RCC), MARCKS phosphorylation is positively correlated with tumor grade, and increased MARCKS expression promotes tumor growth and angiogenesis in vivo in an RCC xenograft model [52]. Suppression of MARCKS by genetic and pharmacologic approaches in high-grade RCC cell lines in vitro decreases cell proliferation and migration and suppresses angiogenesis in vivo by downregulating the AKT/mTOR pathway and HIF-target genes, notably vascular endothelial growth factor-A [52]. MARCKS phosphorylation also promotes cell migration and invasion in vitro and predicts shorter survival times in cholangiocarcinoma patients [53]. Moreover, knockdown of MARCKS in human hepatocellular carcinoma cells in vitro reduces cell migration and invasion, but not cell proliferation [54]. Finally, MARCKS phosphorylation drives motility and invasiveness of melanoma cells of both murine and human origin. Inhibition of MARCKS phosphorylation with a MARCKS-inhibitory peptide abolishes WNT5A-mediated melanoma cell invasion [8,25], suggesting that MARCKS is a crucial promoter of metastasis in melanoma and a candidate anti-metastatic target in melanoma patients.

MARCKS expression and phosphorylation are not universally associated with promoting cancer progression in other solid tumors. For instance, in a mouse colon cancer model, MARCKS depletion reduces motility and invasion in vitro and significantly inhibits metastases in a syngeneic model of colon metastasis in vivo [55]. However, the inactivation of MARCKS is commonly observed in human colon cancers and associated with adverse patient outcomes, suggesting that MARCKS acts as a suppressor of progression in human colorectal cancer [56]. In glioblastoma multiforme (GBM), down-regulation of MARCKS expression with small interfering RNA in cells constitutively expressing EGFRvIII, a mediator of MARCKS phosphorylation, leads to decreased cell adhesion, spreading, and invasion in vitro [57]. However, MARCKS protein expression levels are inversely correlated with GBM proliferation and intracranial xenograft growth rates in vivo, and high expression levels are associated with improved patient survival [58]. These seemingly inconsistent results could be due to the critical role of phosphorylation in the regulation of MARCKS, such that protein expression levels alone fail to correlate with important clinical and biological outcomes. It is well established that phosphorylation of MARCKS, and not MARCKS expression levels, drives cancer cell proliferation and motility. High protein levels of non-phosphorylated MARCKS could attenuate the metastatic phenotype by sequestration of PIP2 at the cell membrane, thereby suppressing PIP2-mediated signaling through downstream pathways such as PI3K/AKT and PLD [15]. 

Conflicting results on the role of MARCKS in cancer progression and metastasis have also been observed in prostate cancer. Dorris et al. showed that knockdown of MARCKS in PC3 cells significantly decreases migration and invasion through downregulation of MMP9 gene expression [6], suggesting that MARCKS promotes prostate cancer metastasis. In agreement with these findings, increased expression of MARCKS is associated with recurrence following surgery for clinically localized prostate cancers [6]. However, Li et al. reported that upregulation of MARCKS protein expression by knockdown of miR-21, a direct regulator of MARCKS, inhibits cell motility and invasion in PC3 cells [59]. In addition, two independent studies demonstrated that targeted knockdown of MARCKSL1, a homologue of MARCKS, in PC3 cells promotes cell migration in vitro [13,60]. While the inhibitory effects of miR-21 knockdown could be due to downstream targets other than MARCKS, they could also be caused by sequestration of PIP2 by unphosphorylated MARCKS, leading to suppression of PIP2-mediated signaling. Indeed, Björkblom et al. showed that dephosphorylated MARCKSL1 increases cell migration, while MARCKSL1 phosphorylated by JNK inhibits cell migration by bundling and stabilizing F-actin [13,38]. Finally, a recent proteomic analysis of urinary and tissue-exudative extracellular vesicles has demonstrated that MARCKS and MARCKSL1 are significantly upregulated in bladder cancer patients [61], and phorbol 12-myristate 13-acetate (PMA)-induced hyperphosphorylation of MARCKS inhibits invasiveness in bladder cancer cells by modulating the cytoskeletal structure [62]. 

**Figure 2:**
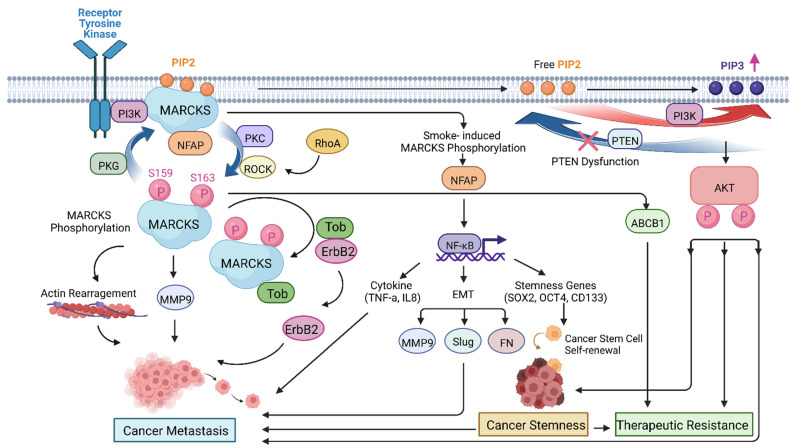
MARCKS participates in mediating target signaling pathways to drive malignant phenotypes that lead to cancer metastasis (through actin rearrangement, MMP9, ErbB2, NF-κB, and AKT pathways), cancer stemness (through the NF-κB pathway), and therapeutic resistance (through AKT, ABCB1, and stemness genes—SOX2, OCT4, and CD133).

Taken together, the majority of the evidence points to a promoting role of MARCKS in cancer metastasis through multiple signaling pathways (Figure 2) in most solid tumors, indicating MARCKS may serve as a potential therapeutic target to tackle metastatic disease. Further studies are needed to define the role of MARCKS in cancer metastasis in cancers where inconsistent results have been observed. Table 1 summaries the roles of MARCKS and MARCKSL1 in different solid tumors.

## 5. MARCKS in Cancer Stemness

MARCKS and MARCKL1 are both substrates of PKC [12], and PKC is an important signalling pathway for stemness, self-renewal, and tumorigenesis [64,65]. For instance, in lung tumor-initiating cells (TICs), protein kinase C iota (PKCiota) is required for oncogene-induced tumor cell expansion and transformation [66], likely through phosphorylation of the ELF3 transcription factor and induced expression of NOTCH3, resulting in cancer stemness and promoting lung cancer development [67]. In breast cancer, protein kinase C α (PKCα) activation leads to the formation of cancer stem cells from non-stem cells, and a PKCα inhibitor depletes stem-like cells [68]. Tobacco smoke-induced phospho-MARCKS upregulates the expression of pro-inflammatory cytokines, causes the epithelial-to-mesenchymal transition and induces stem-like properties in smoke-related lung cancer, which can be reversed by a MARCKS-inhibiting peptide [44]. Furthermore, MARCKS transcript levels are upregulated in chronic myelogenous leukemia quiescent stem/progenitor cells [69], and secreted MARCKS protein has been identified in pancreatic cancer stem cells [70]. Finally, increased levels of MARCKS and MARCKSL1 protein mediated by a long noncoding RNA Zic family member 2 (lncZic2) and transcriptional factor BRG1 were detected during hepatocellular carcinogenesis and hepatic TIC self-renewal [71]. This association suggests that the lncZic2–BRG1–MARCKS/MARCKSL1 signaling cascade might be a potential pathway to target to eliminate hepatic TICs.

## 6. MARCKS in Cancer Therapeutic Resistance

The development of resistance to cancer therapies, including conventional chemotherapeutic agents and radiation, is one of the main causes of cancer relapse leading to mortality [72]. Substantial evidence implicates cancer stem cells and acquisition of a cancer stem cell phenotype in driving therapy resistance [73,74,75]. Given the growing body of evidence demonstrating that MARCKS plays an important role in cancer stemness, it is possible that MARCKS expression and phosphorylation play an important role in therapeutic resistance. Indeed, several studies have demonstrated that MARCKS expression levels are correlated with response to radiation and chemotherapy in multiple cancers. MARCKS is upregulated in oxaliplatin-resistant pancreatic cancer cells and tamoxifen-resistant breast cancer cells compared to cells sensitive to those therapies [46,76]. Moreover, treatment with a MARCKS-inhibiting peptide suppresses lung cancer growth and metastasis in vivo and enhances the sensitivity of erlotinib in lung cancer cells, particularly those tumors with sustained activation of phosphoinositide 3-kinase/AKT signaling [63]. Finally, inhibition of MARCKS phosphorylation sensitizes colon cancer cells to doxorubicin or 5-FU-based chemotherapy by decreasing ATP-binding-cassette transporter family member ABCB1 internalization [77], thereby reducing ABCB1 activity, a major cause of chemotherapy resistance in cancer [78,79]. These studies demonstrated the potential of targeting MARCKS signaling as a novel therapeutic strategy to inhibit cancer stemness and overcome resistance to cancer therapies. However, it is not clear that inhibition of MARCKS could circumvent therapeutic resistance in all cancers. In glioblastoma multiforme (GBM) model systems, knockdown of MARCKS is associated with increased resistance to radiation by increasing DNA repair in PTEN-null GBM cells in vitro and orthotopic xenografts in vivo [58,80]. Additional studies will be necessary to characterize the roles and mechanisms of MARCKS in therapeutic resistance across cancer types.

## 7. Targeting MARCKS as a New Therapeutic Strategy

MARCKS function depends on its NMD and PSD or ED, which are required for its membrane localization and phosphorylation; therefore, peptides targeting these domains have been developed to inhibit its function in various cancers. MANS peptide that targets NMD has been shown to reduce lung cancer metastasis while leaving tumor growth unaffected in vivo [42]. In addition, treatment of breast cancer xenografts with MANS peptide sensitizes cancer cells to paclitaxel and decreases angiogenesis/metastasis of cancer cells by reducing phospho-MARCKS levels [49]. 

Moreover, MANS peptide is able to suppress WNT5A-induced melanoma cell invasion in vitro by inhibiting MARCKS phosphorylation without affecting MARCKS expression [8]. BIO-11006, an analog of the MANS peptide containing the active site of MANS (the first 10 amino acids), is superior to MANS as an anti-cancer agent in that it is smaller in size and more soluble, while maintaining identical MARCKS-inhibitory actions [81]. In a phase II clinical trial of late-stage non-small cell lung cancer, BIO-11006 significantly increased the overall response rate of patients to standard-of-care chemotherapy by decreasing MARCKS phosphorylation [45].

Interestingly, studies have shown that peptides targeting PSD are more efficacious as anti-cancer therapeutics than peptides targeting the myristoylation domain. MPS peptide, a 25-mer peptide targeting PSD, not only suppresses lung cancer metastasis, but also inhibits tumor growth in vivo by decreasing levels of phospho-MARCKS, phosphatidylinositol (3,4,5)-triphosphate, and AKT activity [63]. In addition, MPS peptide suppresses smoke-mediated NF-κB signalling activity, pro-inflammatory cytokine expression, aggressiveness and stemness of lung cancer cells in vitro [44]. In kidney cancer, MPS peptide reduces cell proliferation, migration, and survival and sensitizes cells to regorafenib treatment through inhibiting the AKT and mTOR pathways [52]. In multiple myloma (MM), MPS peptide displays dose-dependent cytotoxicity toward bortezomib-resistant MM cells as a single agent both in vitro and in a xenograft model of MM and sensitizes these cells to bortezomib in combination therapy [82]. In GBM, MARCKS ED peptide produces rapid cytotoxicity through a GBM-specific mechanism involving plasma membrane targeting and intracellular calcium accumulation [83]. Finally, a highly basic 24-amino-acid peptide targeting MARCKSL1 ED was shown to inhibit MARCKSL1 hydrolysis. When synthesized together with an N-terminal HIV-1 Tat transduction domain (TD), MARCKSL1 ED peptide efficiently enters both macrophages and parasites in a Tat TD-dependent manner, suggesting such a strategy may be useful in enhancing cell permeability of MARCKS peptide inhibitors [84].

## 8. Conclusions

MARCKS, a major substrate of PKC, plays a critical role in cancer development and progression and is strongly implicated in cancer metastasis, cancer stemness, and therapeutic resistance (Figure 2). Phosphorylation of MARCKS by PKC leads to MARCKS protein translocation from the plasma membrane to cytosol, where it functions to modulate the cytoskeletal structure and promote cell migration, invasion, and metastasis in the majority of solid tumors. Conflicting results in a few cancer types on the contribution of MARCKS expression levels to metastasis, clinical outcomes, and therapeutic resistance are likely explained by differences in MARCKS phosphorylation, which is primarily responsible for MARCKS regulation and its cellular functions. MARCKS and MARCKS-like proteins promote cancer stemness and resistance to cancer therapies, demonstrating the potential for MARCKS-targeted therapy as a novel therapeutic strategy to inhibit cancer metastasis and overcome resistance to cancer treatment.

## Figures and Tables

**Table 1:** The roles of MARCKS and MARCKSL1 in different solid cancers.

Cancer Type [Reference]	Role	Models	Proliferation /Apoptosis	Migration /Invasion	Tumor Growth	Metastasis	Survival/Grade/Stage	Treatment Resistance	Target
NSCLC [42]	Pro	cell line, xenograft, and TMA		↑		↑	higher grade		E-cad, pAKT, pPI3K, and Slug
NSCLC [41]	Pro	TMA				↑	higher stage		
NSCLC [63]	Pro	cell line, xenograft, and TMA	proliferation ↑ /apoptosis ↓		↑	↑	shorter survival	erlotinib	pAKT
NSCLC [45]	Pro	clinical trial	BIO-11006 (MARCK inhibitor) plus carboplatin showed a less disease progression and a higher response rate compared to carboplatin alone.						
LC [43]	Pro	cell line, TMA, and TCGA	proliferation ↑ /apoptosis ↓				shorter survival	radiation	
LC [44]	Pro	cell line, xenograft, and TMA	proliferation ↑	↑			shorter survival		NF-κB, EMT, and stemness
LSCC [40]	Pro	TMA					shorter survival		
BC [46]	Pro	cell line and TMA	proliferation ↑	↑			shorter survival	tamoxifen	
BC [47,48]	Pro	TMA					shorter survival		
BC [49]	Pro	cell line, xenograft, and TMA	proliferation ↑ /apoptosis ↑	↑	↑	↑	shorter survival	paclitaxel	angiogenic factors
BC [50]	Pro	cell lines	proliferation ↑						ErbB2
RCC [52]	Pro	cell line, xenograft, and TMA	proliferation ↑	↑	↑		higher grade	regorafenib	AKT, mTOR, VEGF, and MM9
CCA [53]	Pro	cell line, xenograft, and human tissue		↑		↑	shorter survival		
HCC [54]	Pro	cell line		↑					
CC [55]	Pro	cell line and xenograft		↑		↑			AURKB
CC [56]	Sup	cell line and TMA	apoptosis ↑				longer survival		TRAIL and AKT
GBM [58]	Sup	cell line, xenograft, clinical trial, and TCGA	proliferation ↓		↓		longer survival		
PCa [6]	Pro	cell line and TMA		↑			more recurrence		
PCa [59]	Sup	cell line		↓					
BC, PCa [60] *	Sup	cell line and xenograft		↓	↓				E-cad, b-catenin, and APC
PCa [13] *	Sup	cell line		↓					
BlaC [62]	Sup	cell line		↓					

Abbreviations: NSCLC, non-small cell lung cancer; LSCC, lung squamous cell carcinoma; LC, lung cancer; BC, breast cancer; RCC, renal cell carcinoma; CCA, cholangiocarcinoma; HCC, hepatocellular carcinoma; CC, colon cancer; GBM, glioblastoma multiforme; PCa, prostate cancer; BlaC, bladder cancer; Pro, promotion; Sup, suppression; * studies on MARCKSL1. ↑, increase; ↓, decrease.
